# Natto (fermented soybeans)-induced anaphylaxis in a surfer with the possibility of sensitization to poly(γ-glutamic acid) from cutaneous exposure to jellyfish sting: a case report

**DOI:** 10.1186/s12245-024-00617-1

**Published:** 2024-03-18

**Authors:** Ayami Shigeno, Tsuyoshi Suzuki, Masakazu Obayashi, Kei Asada, Satoru Matsushima

**Affiliations:** 1 Department of Emergency and Critical Care Medicine, Chutoen General Medical Center, 1-1 Shobugaike Kakegawa Shizuoka, Shizuoka, 436-8555 Japan; 2 Department of Dermatology and Skin Oncology, Chutoen General Medical Center, Shizuoka, Japan

**Keywords:** Natto, Allergy, Poly(γ-glutamic acid), Jellyfish, Cutaneous exposure

## Abstract

**Background:**

We report a case of anaphylaxis induced by natto (fermented soybeans) allergy that occurred following dermal sensitization from a jellyfish sting.

**Case presentation:**

A 49-year-old male presented to the emergency room complaining of an acute onset of erythema with pruritis that appeared while he was surfing. Given that his heart rate dropped to ~ 40 bpm without a decline in blood pressure or oxygen saturation, we suspected anaphylaxis and administered 0.5 mg of adrenaline intramuscularly. Immediately after the muscular adrenaline injection, his heart rate recovered to ~ 60–70 bpm.

**Conclusions:**

The major allergen that induces natto allergy is poly(γ-glutamic acid) (PGA), which is present in its mucilage. Given that PGA is also produced by jellyfish tentacles, it can be inferred that the PGA sensitization occurred via dermal exposure to jellyfish PGA. This is an example of a food allergy induced by animal stings. As PGA is a high-molecular-weight polymer, natto allergy, despite being IgE-mediated, often presents with late-onset anaphylaxis, which typically develops half a day after digestion. PGA has a wide range of applications in pharmaceuticals, cosmetics, and foods. Patients may develop allergic symptoms and experience repeated anaphylaxis with no known cause. Therefore, it is important to obtain a detailed medical history and individually instruct patients suspected of being allergic to PGA to avoid PGA-containing products.

## Background

Natto, a basic ingredient in Japanese food, is a fermented food made from soybeans fermented by *Bacillus subtilis natto*. Recently, it has been reported that natto (fermented soybeans) can cause a delayed, immediate-type allergy [1]. Although immediate-type allergies usually trigger symptoms within a few minutes to an hour after ingestion of the causative substance, symptoms of natto allergy occur several hours to half a day after ingestion, making it difficult to identify the causative substance from a regular medical interview [2]. A previous study has revealed that nearly 90% of patients diagnosed with natto allergy had a hobby in marine sports, and some kind of association was suspected [3]. The major causative allergen in natto that induces allergy is poly(γ-glutamic acid) (PGA) contained in its mucilage. Given that jellyfish tentacles also produce PGA, dermal exposure to jellyfish PGA could induce PGA sensitization [4]. Natto allergy, stemming from jellyfish stings, is an important food allergy associated with animal stings. Surfers who venture into the ocean year-round must be aware of the possibility of dermal sensitization to PGA due to repeated jellyfish stings.

## Case presentation

A 49-year-old male without any substantial food or drug allergy history or other medical history presented to the emergency room (ER) with an acute onset of erythema and pruritus that appeared during surfing. He started surfing at 5 a.m. and noticed urticaria at approximately 6:30 a.m. He subsequently left the ocean and headed to work. After arriving at work, he developed respiratory distress at approximately 7 a.m. and called for emergency medical assistance. He arrived at our ER at approximately 8 a.m., and his vital signs were as follows: heart rate (HR), 95 bpm; blood pressure (BP), 124/79 mmHg; oxygen saturation (SpO_2_), 97% (ambient air); respiratory rate, 20 cycles/min; body temperature, 36.4℃; and Glasgow Coma Scale, E4V5M6. Multiple erythematous plaques with a tendency to merge were observed on extremities and trunk. These skin findings are consistent with those of urticaria. He complained of pruritic erythema and respiratory distress; however, no airway obstruction sounds were heard during auscultation. Approximately 10 min after arrival at the ER, he complained of nausea, and his HR dropped to ∼40 bpm without any decrease in BP or oxygen saturation. We suspected anaphylaxis and administered 0.5 mg of adrenaline intramuscularly. Immediately after the intramuscular adrenaline injection, his HR recovered to approximately 60–70 bpm. A complete blood cell count, basic metabolic panel, and electrocardiogram were performed, and all results were within normal limits. The patient was diagnosed with allergy-associated anaphylaxis and was admitted to the emergency care unit for observation. Two hours after admission (4 h after onset), erythema persisted, and the patient also had nasal obstruction; therefore, an additional 0.5 mg of adrenaline was administered intramuscularly. Following gradual symptom improvement, the patient was discharged the following day.

**Fig. 1:**
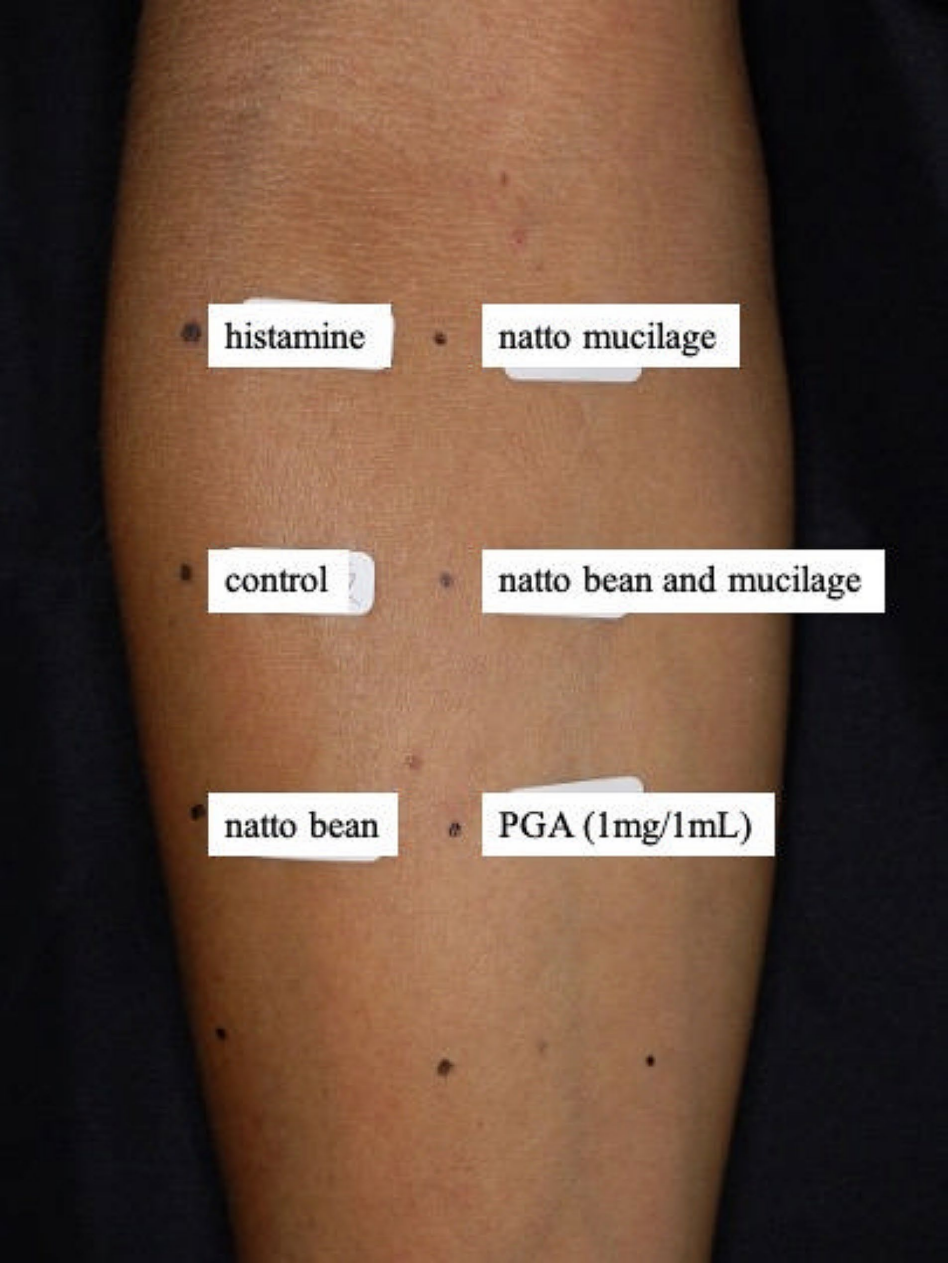
The prick test (Histamine, a control solution, natto bean (fermented soybean), natto mucilage, a mixture of natto bean and mucilage, and PGA were applied to the inner forearm of the patient)

An additional medical history interview was conducted, revealing that the patient had consumed natto (fermented soybeans) at dinner the previous night at approximately 6 p.m. Given that several previous case reports have indicated that surfing could be a risk factor for natto allergy and late-onset anaphylaxis, [3] we planned another hospitalization to perform natto and PGA prick tests. On day 8 after discharge from the first admission, the patient was readmitted for a prick test to reveal the association between natto and allergic reactions. Histamine, a control solution, natto bean (fermented soybean), natto mucilage, a mixture of natto bean and mucilage, and PGA (1 mg/1 mL) were applied to the inner forearm of the patient (Fig. 1), and a prick test was performed.

**Fig. 2:**
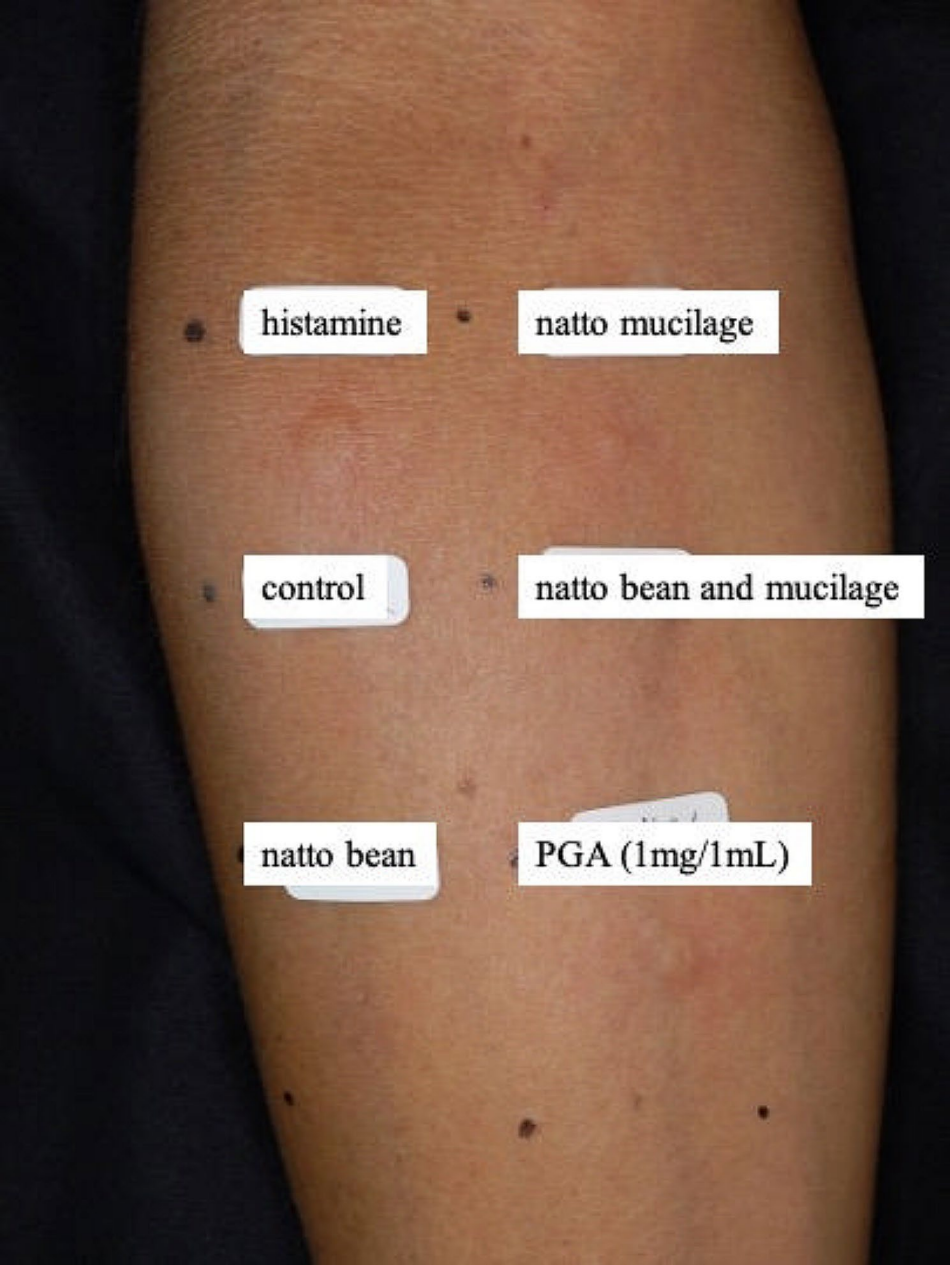
Results of the prick test (Histamine, a control solution, natto bean (fermented soybean), natto mucilage, a mixture of natto bean and mucilage, and PGA)

Fifteen minutes after initiating the prick test, histamine, natto mucilage, and PGA induced erythema and wheals (Fig. 2). The results of the skin prick test are shown in Table 1. The patient was discharged the following day without an anaphylactic reaction. He was diagnosed with PGA allergy, advised to avoid natto, and prescribed an EpiPen.

**Table 1:** Results of skin prick test

	Erythema (mm)	Puffiness (mm)
Histamine	15*15	9*8
Control	-	-
Natto Bean	-	-
Natto Mucilage	7*8	5*4
PGA	20*10	10*6

## Discussion and conclusions

Conventionally, it is considered that food allergy sensitization to orally ingested food antigens is established primarily via the enteric route [5]. In 2008, Lack et al. proposed the dual-allergen exposure hypothesis and discussed the importance of transcutaneous sensitization in food allergies [6]. Based on this hypothesis, tolerance can occur as a result of oral exposure to food, and allergic sensitization results from cutaneous exposure [6]. 

Considering a food allergy induced by animal bites, Commins et al. reported an allergy to mammalian red meat caused by tick bites [7]. In addition, Chung et al. reported a high incidence of cetuximab-induced anaphylaxis in certain regions of the United States [8]. Cetuximab is a monoclonal antibody that binds to the skin growth factor receptor and inhibits the function of the epidermal growth factor receptor. Chung et al. found that cetuximab-specific IgE reacts with the oligosaccharide galactose-α-1,3-galactose (α-gal) [8]. Commins et al. revealed that α-gal was also the cause of delayed anaphylaxis after ingestion of animal meat and further reported that the production of IgE antibodies toward α-gal was associated with tick bites [9]. A positive correlation was detected between mammalian red meat-specific IgE levels and cetuximab-specific IgE levels. Given that tick saliva contains a variety of different proteins, its sensitization results in the production of IgE antibodies against oligosaccharide α-gal found in mammalian red meat. This is a proposal for a new category of food allergies induced by animal stings [7, 8]. 

Although natto allergy is an IgE-mediated allergy, it often presents with late-onset anaphylaxis, developing approximately half a day after digestion. Natto causes delayed anaphylaxis because the natto allergen, PGA, is a high-molecular-weight polymer that is slowly absorbed as it gradually dissolves in the intestinal tract after ingestion [1, 2]. The major allergen in natto is PGA, a viscous component of natto that is utilized in a wide range of fields, including pharmaceuticals, cosmetics, and foods. Moreover, given that PGA is produced in jellyfish tentacles, the sensitization route of PGA is presumed to be transcutaneous exposure to jellyfish PGA at the time of a jellyfish sting [10]. Jellyfish, which are cnidarians, produce PGA in their tactile cells when the target touches their tentacles, and the osmotic pressure regulation effect causes the stinger to puncture the target [4, 11]. A previous study has shown that nearly 90% of patients with natto allergy have a history of marine sports [3]. Past reports have also highlighted that natto allergy is dose-dependent and that the onset of natto allergy is dependent on the amount of natto ingested [1, 12]. 

In the current case report, the patient ate natto for dinner at approximately 6 p.m. the day before surfing. He mentioned that he did not have a daily habit of eating natto and had consumed natto after a prolonged interval.

The patient surfed from approximately 5 a.m. the next morning and noticed urticaria all over his body at approximately 6:30 a.m. The patient had been surfing as a hobby for over 20 years since his youth and had been stung by jellyfish countless times in the past. He had never experienced any allergic symptoms after eating natto, and this was the first time he had been diagnosed with a natto allergy.

Natto allergy, stemming from jellyfish stings, is an important food allergy associated with animal stings. It is essential for surfers who enter the ocean year-round to be aware of the possibility of dermal sensitization to PGA due to repeated jellyfish stings. Therefore, surfers with repeated exposure to jellyfish in the ocean are considered to be at an increased risk of natto allergy [3, 13]. Patients can develop allergic symptoms and experience repeated anaphylaxis of no known cause. It is crucial to obtain a detailed medical history and individually instruct patients suspected of being allergic to PGA to avoid the use of PGA-containing products.

## Data Availability

Not applicable.

## Abbreviations

PGA: poly(γ-glutamic acid)
ER: emergency room
HR: heart rate
BP: blood pressure
